# Disability and labour market participation among smallholder farmers in Western Kenya

**DOI:** 10.1371/journal.pone.0306458

**Published:** 2024-07-05

**Authors:** Stevens Bechange, Emma Jolley, Anita Jeyam, George Okello, Ben Wekesa, Elena Schmidt

**Affiliations:** 1 Sightsavers Kenya Country Office, Nairobi, Kenya; 2 Sightsavers United Kingdom, Haywards Heath, United Kingdom; 3 Innovations for Poverty Action (IPA) Kenya, Nairobi, Kenya; University of Sialkot, CHINA

## Abstract

**Background:**

Despite the importance of labour market participation and the high number of people with disabilities in rural Africa who rely on subsistence agriculture to survive, very few studies have documented labour market outcomes among farmers with and without disabilities in Africa.

**Objective:**

We examined how labour market participation differed by disability and other factors among smallholder farmers in Western Kenya.

**Methods:**

We use cross-sectional data collected between January and April 2022 from sorghum farmers enrolled in a trial evaluating the impact of a programme designed to improve labour market participation among sorghum farmers in rural Western Kenya. Disability and Labour market outcomes were assessed using questions from the Washington Group /ILO Labor Force Survey Disability Module the ILO Labour Force Survey module respectively. Univariate and multiple regression analyses were conducted to identify socio-demographic characteristics and other related factors associated with labour market participation.

**Results:**

Among 4459 participants, disability was reported by 20.3% of women and 12.3% of men. Labour market participation was reported by 77.1% and 81.3% of women and men, respectively. Adjusting for demographic confounders, having a disability was associated with a lower likelihood of labour market participation (odds ratio 0.59, 95% confidence interval, 0.42–0.83, P = 0.001). These findings were similar in a modified model that looked at functional difficulties separately from anxiety and depression. Women, older participants, and those who were dependent on others were also more likely not to report participation in the labour market.

**Conclusions:**

Increased recognition and understanding of functional limitations among smallholder farmers is vital for the success of economic empowerment programmes aimed at increasing labour market participation among the most vulnerable populations.

## Introduction

A majority of rural households in sub-Saharan Africa continue to rely on small-scale agriculture as their primary source of income [1, 2]. Measures that seek to improve participation and access to higher-value markets are increasingly seen as a vital form of social protection against the harsh economic realities of asset and income poverty and vulnerability. Across much of sub-Saharan Africa, recent policy interventions have stressed the critical need to increase market participation and linkages between rural producers and urban consumers [3], value addition [4], and enhancing access to credit for small scale farmers who are most vulnerable, including those with disabilities [5]. Disability, broadly defined in this paper to be consistent with the United Nations Convention on the Rights of People with Disabilities (UNCRPD) definition [6], is a complex and multi-dimensional concept [7]. The contextual nature of disability emphasized by the UNCRPD is useful for understanding the relationship between disability and labour market participation among smallholder farmers. Empirical data on disability in the agriculture sector in Kenya is scarce. However, prevalence of disability in rural parts of the country, where the majority of agricultural workers reside, is estimated to be twice as high as in urban locations [8].

Farmers with disabilities are often identified as being at greater risk of lower, or unfavorable participation in the labour market [9]. Studies conducted in low- and middle-income settings show that people with disabilities participate in agriculture by contributing their labour (i.e., carrying out household farming activities) but exerting no control over production, marketing or selling decisions [10, 11]. Although the evidence is not always robust, there are a number of systematic, attitudinal, or environmental barriers that have been identified to limit the participation of farmers with disabilities in agriculture and access to higher-value markets. These include misconceptions that people with disabilities cannot engage in productive farming activities [12], distrust by financial institutions that excludes them from accessing credit facilities [13], inaccessible training and infrastructure, lack of access to land and tools, psychosocial difficulties and self-exclusion from the labour market due to self-stigma [14, 15]. Compared to low- and middle-income countries (LMICs), fewer people with disabilities are engaged in smallholder farming in high-income countries.

Previous studies conducted in high-income countries broadly agree that disability has a negative impact on labour market prospects for people with disabilities [16–20]. Within this literature, there are two streams of work. One set of studies highlight tensions between existing policies and practices around active citizenship and participation in the labour market for people with disabilities [21–24]. To some degree, these tensions reflect the increased awareness and attention to the social and employment rights of people with disabilities in most high-income countries. These studies tend to emphasize workplace inclusion and the performance of national systems—the practices, legislation and policies that have been put in place by governments to help people with disabilities to find and remain in gainful employment [25]. They pay less attention, however, to the capacities and everyday practices of people with disabilities to secure and maintain gainful employment. A second set of previous studies conducted in high-income countries reveal more complex and mixed findings, with gender inequality and unfavorable employment conditions persisting across countries and continuing to act as a barrier to labour market participation for people with different types of disabilities [26–28]. An earlier study in Norway found that young men with disabilities experienced more extreme labour force participation disadvantages than young women with disabilities [26], which is surprising, given the substantial literature from other high-income countries which show that women with disabilities experience more labour market participation inequalities than men with disabilities [29].

While these studies have significantly advanced our understanding of labour market participation and associated dimensions of disadvantage, there is still very limited research from resource-limited settings that explores the relationship between disability and labour market outcomes [30, 31]. Although there are some published analyses using data from labour force surveys in these countries, these do not specifically relate to people with disabilities [32]. Interventions designed to adress labour market participation for smallholder farmers with disabilities in Kenya or other LMICs, for example, have not been well researched [33], and it is not clear how such interventions need to be adapted [9]. Moreover, compared to high income countries, more people with disabilities are engaged in smallholder farming in low- and middle-income countries (LMICs). The only published study from LMICs that explores the relationship between disability and labour market outcomes is a household survey in Indonesia [34]. There is no published quantitative analysis of this relationship among smallholder farmers in sub-Saharan Africa.

To address the gaps in knowledge and inform disability inclusion policies and practices, there is a continuing need to better understand and respond to the barriers that exclude smallholder farmers with disabilities from participating in the labor market in different contexts. This information can improve the design and implementation of economic empowerment programmes aimed at increasing labour market participation among the most vulnerable populations, and inform strategies for achieving decent employment for people with disabilities and promoting disability inclusive development. Kenya provides an interesting setting to examine these issues not only because of the suitable labor market context [35] but also due to the leadership and commitment that the government of Kenya has shown on disability rights in recent years [36]. To better understand the relationship between socio-demographic factors, labour market participation, and disability, we draw on perspectives from the international labour organization (ILO) [37] and the UNCRPD [6] in our analysis of disability and its association with labour market participation among sorghum farmers in rural Kenya.

To the best of our knowledge, no previous study has explored the relationship between disability and labour market participation for smallholder farmers in Kenya. Therefore, in this paper, we sought to i) describe the prevalence of disability and labour market outcomes within a representative sample of smallholder farmers in western Kenya, and ii) assess the factors associated with labour market participation in this sample. Aspects of mental health are also analysed because they overlap with or derive from disability. We hypothesized that having a disability would be associated with lower levels of participation in the labour market, with those who come from relatively poorer households, women and older people having the lowest levels of participation. We also hypothesized that mental health conditions, specifically depression and anxiety, would moderate the relationship between having a disability and labour market outcomes.

## Methods

### Study design and setting

Between January 2022 and April 2022 we enrolled individuals in a non-randomized cluster field trial to evaluate the impact of a 5-year programme [38] designed to improve labour market participation through inclusive farming interventions targeting smallholder sorghum farmers in rural Western Kenya. This paper used data from the baseline visit of this field trial. The study catchment area covers a 200 km radius around Migori, Homa Bay, Kisumu, Siaya and Busia counties. Subsistence agriculture is the main livelihood activity in the area—although various forms of trade and fishing are important activities for some sectors of the population. The majority of people in the area have not received education beyond primary school level. To be eligible, participants must have been 18 years or older, residents of one of the selected sub-counties, self-report that they are currently farming sorghum or have access to land for farming and willing to consider sorghum farming for sale, and able to provide informed consent.

### Study sample

The sample for the study was drawn from 14 sub-counties in Western Kenya. These included 7 intervention and 7 control sub-counties included in the field trial. Intervention sub-counties were determined by the location of the intervention project. Seven control sub-counties were selected from non-adjacent areas and socio-demographically matched (e.g., with respect to key population characteristics). Two-stage cluster sampling was used to sample participants, where primary sampling units (clusters) were selected probability proportional to size using the 2019 Population and Housing Census [8], and then households in selected clusters were sampled by random walk. Within households, all eligible adults were offered participation. The sample comprised 4,459 individuals.

### Study variables and measures

The survey tool included a number of modules constructed based on existing validated tools.

#### Socio-demographic characteristics

We collected data on household size, relative wealth, participant sex, age, marital status, religion, education, and main source of income. Income was categorized as “trade” for all forms of activity that created income from selling (e.g., craft or market vendors); “salary” for wage and salaried employment; “farming” for agricultural farming or fishing; and “dependent” for those who reported receiving money from others as their main source of income.

#### Labour market outcomes

Labour market outcomes were assessed using questions from the Washington Group/ International Labor Organization (ILO) Labor Force Survey module, Agriculture work start version. Variable derivation is summarized below, readers should refer to the related ILO variable derivation guide for full details [39]. Individuals were defined as employed/engaged in the labour market if they had been “engaged in any activity to produce goods or provide services in exchange for pay or to generate profit” [39], during the week preceding the survey. This was derived from responses on questions related to intended destination of produced goods, non-agricultural work, and absence from work. We used the expression “engaged in the labour market” throughout the paper to refer to employed persons in order to facilitate an intuitive understanding of the term.

Individuals were classified as employers, independent workers without employees, dependent contractors, employees or contributing family workers depending on their self-reported type of employment, but also their responses to other questions including decision-making, type of pay, independent price setting. Nature of main job was defined as formal or informal depending on the type of employment, responses to questions including social protection status, the bookkeeping process, registration status of the business. Time-related underemployment was defined as working less than 40 hours per week and available and wanting to work more.

#### Disability

Disability status was assessed using questions on self-reported functional difficulty included in the Washington Group /ILO Labor Force Survey Disability Module (LFS-DM). The module includes eight questions about functional difficulties in performing basic body functions. For six domains (seeing, hearing, walking, concentrating, caring for oneself, and communicating), responses are measured on a 4-point scale from ‘no difficulty’ to ‘cannot do at all’. Responses to the questions on anxiety and depression are measured on a-5-point scale indicating frequency of experiencing from ‘never’ to ‘daily’. *Disability* was defined based on the response of “a lot of difficulty” or “cannot do at all” to at least one of the six questions on the former six domains, or “daily” to at least one of the questions on anxiety and depression.

### Data collection procedures

At enrollment, participants were interviewed regarding socio-demographic characteristics, disability, and labour market participation using a standardized questionnaire. Research assistants trained in survey administration, rapport-building techniques and approaches for eliciting information on sensitive topics, conducted face to face structured interviews with participants and recorded data using tablet devices running SurveyCTO software. Research assistants were proficient in Swahili and at least one other local language spoken in the study area. The completion of the questionnaire took 40 to 60 minutes per participant. Participants were given the choice of where the interview would take place; often an open area in the homestead (e.g., under a tree) was preferred for greater confidentiality. Research assistants received extensive training on confidentiality protocols. Interviewers and participants were matched by ethnic background. The questionnaires were translated into three local languages (Swahili, Luo, and Luhya) and independently back-translated into English. Supervisors checked the quality of data through random re-survey of households and daily verification of all submitted survey data.

### Statistical analysis

Data were managed and analyzed using R v4.3.0 [40] and Stata16 [41]. Baseline characteristics were compared across sex using Wald-tests for categorical variables and Wilcoxon tests for continuous variables. Our first aim was to understand the relationship between socio-demographic factors including disability status, and employment outcomes. Age and sex are potential confounding factors for these relationships as many characteristics as well as outcomes are likely to differ across both sex and age. For example, people become more likely to acquire functional difficulties (and hence have a disability) as they become older. They are also less likely to be employed in older age. For the binary employment outcomes of interest, we therefore used univariate logistic regression models, adjusted for age and sex, to explore these relationships. Furthermore, we wanted to understand if there was a gap in employment outcomes between people with and without disabilities once other socio-demographic factors were accounted for. For this purpose, we used a multivariable logistic regression model controlling for sex, age, religion, main source of income, education, marital status, household size, being head of household and presented the adjusted odds-ratios for the association between disability and employment outcomes. Type of occupation was a categorical variable; we therefore used a multinomial logistic regression model to explore the associations with this outcome. The main source of income was not included as a covariate for modelling the type of occupation given that the source of income plays a large part in defining the type of occupation. Finally, we conducted sensitivity analyses to further explore the relationship between disability and engagement in the labour market. We examined whether the association between having a functional difficulty and being engaged in the labour market differed from the more general association between disability (including both affect and functional difficulties), and engagement in the labour market. All modelling and testing accounted for clustering within sublocations households.

### Ethical considerations

The study protocol was reviewed and approved by the National Commission for Science, Technology, and Innovation (NACOSTI) (Ref #: 676151) and the institutional ethics review committee of Strathmore University (SU-IERC) (Ref #: SU-IERC1234/21). Written informed consent to participate in the study was obtained from each participant in one of three local languages (or sign language for participants with hearing impairments) via a digital consent form. All participants were paid an equivalent of US$1.5 each as a token of appreciation of their time.

## Results

### Participant characteristics

Descriptive statistics for key variables used in this analysis are shown in Table 1, for the total sample and also disaggregated for female and male participants. We approached 4,491 adults for study participation. Of these, 32 (0.7%) declined to participate. Thus, 4,459 participants were included in the study, which was a response rate of 99.3%. The median age of study participants was 44 years; 61.9% were female. Majority of the participants were married or cohabiting (77.9%), lived in households with three or more other people (81.5%) and reported being christians (80.4%). Around two-thirds (67.3%) were heads of household. Over 65.5% of participants had primary school as the highest level of education attained. Farming was the main source of income for 63.0% of participants. Distribution of relative wealth in our sample was comparable to that of the general population.

**Table 1:** Characteristics of study participants (N = 4459).

Variable		Female (N = 2758)		Male (N = 1701)		Total		p-value
		n	%	n	%	N	%	
Age (years)	18–30	571	20.7	270	15.9	841	18.9	<0.01
	31–40	725	26.3	399	23.5	1124	25.2	
	41–50	561	20.3	346	20.3	907	20.3	
	51–60	408	14.8	292	17.2	700	15.7	
	>60	493	17.9	394	23.2	887	19.9	
Highest education level	Never attended school	298	10.8	50	2.9	348	7.8	<0.01
	Primary	1870	67.9	1051	61.8	2921	65.5	
	Secondary	497	18.0	441	25.9	938	21.0	
	More than secondary	91	3.3	159	9.3	250	5.6	
Marital status	Single/separated/divorced	43	1.6	86	5.1	129	2.9	<0.01
	Married/co-habiting	1917	69.5	1558	91.6	3475	77.9	
	Widowed	798	28.9	57	3.4	855	19.2	
Religion	Catholic	538	19.5	418	24.6	956	21.4	<0.01
	Protestant	856	31.0	510	30.0	1366	30.6	
	Evangelical	820	29.7	463	27.2	1283	28.8	
	African Instituted Church	527	19.1	277	16.3	804	18.0	
	Other	17	0.6	33	1.9	50	1.1	
Main source of income	Farming	1637	59.4	1173	69.0	2810	63.0	<0.01
	Salary	133	4.8	187	11.0	320	7.2	
	Dependent	188	6.8	38	2.2	226	5.1	
	Trade	653	23.7	195	11.5	848	19.0	
	Other	105	3.8	95	5.6	200	4.5	
	None	42	1.5	12	0.7	54	1.2	
Head of household	Yes	1363	49.4	1638	96.3	3001	67.3	<0.01
Household size	Living alone	87	3.2	45	2.6	132	3.0	0.01
	1–2	436	15.8	257	15.1	693	15.5	
	3–5	1339	48.5	755	44.4	2094	47.0	
	6–8	708	25.7	462	27.2	1170	26.2	
	9+	188	6.8	182	10.7	370	8.3	
Relative wealth	Poorest quintiles (Q1-Q2)	1166	42.3	737	43.3	1903	42.7	0.53
	Wealthier quintiles (Q3-Q5)	1,589	57.7	964	56.7	2,553	57.3	

All baseline characteristics differed significantly between male and female participants, apart from relative wealth. Female participants reported lower levels of formal education and were more likely to be widowed (Table 1). Women were also more likely to report trade as their main source of income, whereas men were more likely to report farming as their main source of income. While the majority of male participants were heads of household (96.3%), this was the case for only half the female participants (49.4%). Men were more likely to be living in larger households.

### Prevalence of disability

Overall, the prevalence of disability was 17.2%, with 9.7% of participants reporting at least one difficulty in the six functional domains (seeing, hearing, mobility, self-care, cognitive, communication) and 9.8% reporting daily anxiety or depression (Table 2). The most commonly reported domains were anxiety (7.1%), mobility (5.7%) and depression (5.3%). The prevalence of disability was significantly higher among female participants than male participants overall, and in both functional difficulty and anxiety/depression. By domain, the prevalence of difficulties in mobility, cognition and self-care were higher among female than male participants.

**Table 2:** Prevalence of disability and domains of difficulty.

	Female		Male		Total		p-value
	N	%	n	%	N	%	
Disability (all)	559	20.3	208	12.3	767	17.2	<0.01
*Functional difficulties*							
At least one functional difficulty	315	11.4	116	6.8	431	9.7	<0.01
Vision	93	3.4	43	2.5	136	3.1	0.15
Hearing	23	0.8	7	0.4	30	0.7	0.12
Mobility	190	6.9	65	3.8	255	5.7	<0.01
Cognitive	85	3.1	11	0.6	96	2.2	<0.01
Self-care	27	1.0	6	0.4	33	0.7	<0.01
Communication	3	0.1	2	0.1	5	0.1	0.93
*Affect*	330	12.0	105	6.2	435	9.8	<0.01
Anxiety	238	8.6	77	4.5	315	7.1	<0.01
Depression	185	6.7	49	2.9	234	5.3	<0.01

### Employment outcomes

Most participants (78.7%) were engaged in the labour market. Among them, the largest group were independent workers without employees (63.2%), followed by employers (18.0%) and employees (15.8%) (Table 3). The vast majority of participants had an informal main job (92.9%) and 61.8% reported wanting to change their current employment situation.

**Table 3:** Description of labour market outcomes.

		Females		Males		Total		p-value
		n	%	n	%	N	%	
Engaged in labour market		2124	77.1	1381	81.3	3505	78.7	<0.01
Nature of main job	Formal	93	4.4	156	11.4	249	7.1	<0.01
	Informal	2026	95.6	1216	88.6	3242	92.9	
Type of employment	Contributing family worker	22	1.0	10	0.7	32	0.9	<0.01
	Dependent contractor	43	2.0	33	2.4	76	2.2	
	Employee	214	10.1	337	24.5	551	15.8	
	Employer	287	13.5	342	24.9	629	18.0	
	Independent worker without employees	1556	73.3	653	47.5	2209	63.2	
Wanting to change current employment situation		1277	60.2	885	64.3	2162	61.8	0.02
Number of days worked in week	1–5	672	31.7	490	35.6	1,162	33.2	0.08
	6 or more	1,449	68.3	886	64.4	2,335	66.8	
Number of hours usually worked weekly	<25	651	30.6	293	21.3	944	27.0	<0.01
	25–39	539	25.4	254	18.4	793	22.7	
	40–59	516	24.3	399	29.0	915	26.1	
	60+	418	19.7	431	31.3	849	24.3	
In time-related underemployment		526	24.8	290	21.0	816	23.3	0.05

All labour market outcomes examined differed between men and women. Compared to female participants, male participants were more likely to be engaged in the labour market, to have a formal job, to be employees or employers whereas female participants were more likely to be independent workers without employees than male participants. Male participants were also more likely to be working more than 60 hours a week, and to want to change their current employment situation.

Results of univariate models (Table 4) showed that participation in the labour market was associated with being male, having a salary, trade or another non-dependent main source of income, and living with six or more other people. Older people, those with disabilities, those who were mainly dependent on others or had no income and those from smaller households, were less likely to be engaged in the labour market. Multivariable models showed that, when all other covariates were adjusted for, the gap between those with and without disabilities remained (OR = 0.59 [0.42, 0.83]). When we looked at functional difficulties separately from anxiety and depression, the relationship between having a functional difficulty and being engaged in the labour market was similar (OR = 0.50 [0.32, 0.78]).

**Table 4:** Factors associated with labour market outcomes—Univariate model results—Data are OR and 95% CI.

	Engaged in labour market	Informal employment	Wants to change employment situation	In time-related underemployment	Working <25 hours per week	Working 6–7 days/week
**Sex** *						
Male vs Female	**1.29 [1.08,1.54]**	**0.36 [0.27, 0.47]**	**1.19 [1.03,1.38]**	**0.81 [0.65,1.00]**	**0.61 [0.51,0.73]**	0.84 [0.69,1.03]
**Age (years)** *	**0.97 [0.96, 0.98]**	**1.01 [1.00, 1.02]**	**0.98 [0.97,0.98]**	**0.99 [0.98,0.99]**	**1.01 [1.01,1.02]**	**1.01 [1.00,1.02]**
**With disability**	**0.59 [0.44, 0.78]**	1.43 [0.87, 2.33]	1.21 [0.92,1.59]	1.04 [0.81,1.34]	0.95 [0.74,1.23]	1.00 [0.82,1.23]
**Education**						
More than secondary	1.49 [0.85, 2.63]	**0.04 [0.01, 0.17]**	1.19 [0.73,1.95]	0.61 [0.36,1.01]	0.65 [0.39,1.07]	**0.34 [0.19,0.58]**
Secondary	1.02 [0.66, 1.57]	**0.12 [0.03, 0.57]**	1.29 [0.86,1.93]	0.96 [0.65,1.43]	1.00 [0.64,1.56]	**0.51 [0.34,0.77]**
Primary	1.34 [0.93, 1.94]	0.26 [0.06, 1.07]	1.37 [0.97,1.92]	0.95 [0.68,1.33]	1.02 [0.72,1.45]	**0.59 [0.41,0.86]**
None	Ref	ref	ref	ref		ref
**Main Source of Income**						
Farming	Ref	ref	ref	ref		ref
Salary	**5.15 [2.44,10.84]**	**0.15 [0.08, 0.26]**	1.35 [0.99,1.84]	**0.54 [0.35,0.82]**	**0.66 [0.48,0.90]**	**0.52 [0.38,0.72]**
Trade	**9.82 [4.90,19.70]**	**0.32 [0.20, 0.52]**	0.99 [0.75,1.31]	**0.53 [0.40,0.71**]	**0.64 [0.47,0.87]**	0.93 [0.67,1.30]
Dependent on others or none	**0.15 [0.10, 0.22]**	0.81 [0.23, 2.85]	1.14 [0.74,1.75]	1.03 [0.59,1.79]	2.24 [1.41,3.58]	0.69 [0.40,1.18]
Other	**3.29 [1.95, 5.56]**	0.63 [0.31, 1.27]	1.26 [0.87,1.84]	**0.42 [0.28,0.62**]	0.73 [0.47,1.14]	0.70 [0.48,1.02]
**Marital status**						
Married/co-habiting	ref	ref	ref	ref	Ref	ref
Divorced/separated/single	0.99 [0.57, 1.72]	1.71 [0.77, 3.80]	1.06 [0.71,1.60]	0.90 [0.53,1.51]	0.94 [0.56,1.59]	1.26 [0.84,1.89]
Widowed	0.92 [0.68, 1.24]	1.80 [0.98, 3.33]	1.05 [0.81,1.36]	0.81 [0.63,1.05]	1.00 [0.75,1.33]	0.83 [0.65,1.07]
**Being head of household**	1.27 [0.99, 1.63]	1.06 [0.68, 1.64]	1.19 [0.98,1.46]	0.92 [0.69,1.23]	**0.69 [0.56,0.83]**	1.12 [0.93,1.34]
**Household size**						
Living with 3–5 people	Ref	ref	ref	ref	Ref	ref
Living alone or with 1–2	**0.75 [0.63, 0.89]**	1.13 [0.71, 1.78]	0.99 [0.78,1.25]	0.95 [0.76,1.18]	**1.54 [1.24,1.90]**	0.85 [0.68,1.07]
People						
Living with 6 or more people	**1.28 [1.02, 1.61]**	0.76 [0.55, 1.05]	1.03 [0.84,1.28]	0.99 [0.84,1.16]	1.16 [0.97,1.40]	0.84 [0.72,0.99]
**Religion**						
Evangelical	ref	ref	ref	ref	Ref	ref
Catholic	1.24 [0.85, 1.80]	1.36 [0.89, 2.10]	0.86 [0.66,1.12]	0.97 [0.77,1.21]	1.16 [0.87,1.55]	1.08 [0.85,1.37]
Protestant	1.03 [0.79, 1.36]	0.80 [0.52, 1.22]	0.85 [0.64,1.13]	0.99 [0.80,1.24]	1.01 [0.76,1.33]	0.87 [0.65,1.18]
African Instituted Church /Other	0.86 [0.61, 1.22]	1.43 [0.90, 2.27]	1.06 [0.79,1.43]	0.86 [0.65,1.15]	1.10 [0.81,1.48]	0.80 [0.60,1.05]
**Relative wealth**						
Wealthier quintiles (vs Q1-Q2)	1.11 [0.89, 1.40]	**0.31 [0.22, 0.44]**	0.83 [0.66,1.05]	**0.76 [0.62,0.94]**	0.93 [0.73,1.17]	0.93 [0.77,1.13]

*Models including sex or age alone—all other models are adjusted for age and sex

Among those engaged in the labour market, having a formal job was associated with being male, higher levels of formal education, those who had a salary or trade as main source of income and those who were from wealthier households. Older people were more likely to have an informal main job than younger people. There was no evidence of significant association between disability status and having an informal job. Multivariable models showed that, when all other covariates were included, the association with disability remained statistically non-significant (OR = 1.00 [0.56, 1.77]). Results remained similar for the association between functional difficulty and having a formal job (OR = 1.24 [0.56, 2.74]) (Table 5).

**Table 5:** Associations with labour market outcomes—Results from multivariable models—Data are OR [95%CI].

	Engaged in labour market	Informal employment	Wants to change employment situation	Time-related underemployment	Works <25 hours per week	Works 6–7 days/week
Sex*						
Male vs Female	1.07 [0.78, 1.46]	0.36 [0.27, 0.47]	1.08 [0.85,1.38]	0.79 [0.59,1.04]		0.76 [0.60,0.96]
Age (years)*	0.97 [0.97, 0.98]	1.01 [0.99, 1.02]	0.98 [0.97,0.98]	0.99 [0.98,1.00]	1.01 [1.00,1.02]	1.01 [1.00,1.02]
With disability	0.59 [0.42, 0.83]	1.00 [0.56, 1.77]	1.20 [0.90,1.60]	1.02 [0.78,1.32]	0.92 [0.70,1.21]	0.95 [0.77,1.18]
Education						
More than secondary	1.77 [0.87, 3.58]	0.06 [0.01, 0.33]	1.34 [0.81,2.21]	0.69 [0.40,1.17]	0.63 [0.38,1.06]	0.33 [0.19,0.57]
Secondary	1.00 [0.59, 1.71]	0.20 [0.04, 0.98]	1.46 [0.95,2.23]	1.01 [0.65,1.57]	1.03 [0.64,1.67]	0.48 [0.31,0.72]
Primary	1.29 [0.83, 2.01]	0.38 [0.08, 1.70]	1.43 [1.00,2.03]	0.98 [0.67,1.43]	1.08 [0.76,1.54]	0.57 [0.39,0.83]
None	Ref	ref	ref	ref	ref	Ref
Main Source of Income						
Farming	ref	ref	ref	ref	ref	Ref
Salary	5.11 [2.34,11.16]	0.17 [0.10, 0.30]	1.38 [0.99,1.92]	0.57 [0.37,0.87]	0.67 [0.49,0.93]	0.56 [0.42,0.77]
Trade	10.33 [5.28,20.23]	0.34 [0.21, 0.55]	1.01 [0.76,1.35]	0.55 [0.41,0.74]	0.64 [0.47,0.86]	0.95 [0.68,1.32]
Dependent on others or none	0.16 [0.11, 0.24]	1.23 [0.32, 4.75]	1.21 [0.77,1.90]	1.07 [0.61,1.87]	2.18 [1.35,3.52]	0.77 [0.44,1.35]
Other	3.50 [2.07, 5.92]	0.63 [0.30, 1.33]	1.24 [0.84,1.81]	0.43 [0.29,0.64]	0.71 [0.46,1.12]	0.71 [0.47,1.06]
Marital status						
Married/co-habiting	ref	ref	ref	ref	ref	Ref
Divorced/separated/single	1.03 [0.56, 1.91]	2.04 [0.70, 5.97]	1.08 [0.71,1.65]	0.95 [0.54,1.66]	0.89 [0.50,1.57]	1.38 [0.86,2.21]
Widowed	0.84 [0.56, 1.26]	1.85 [1.01, 3.40]	0.93 [0.67,1.30]	0.85 [0.61,1.18]	1.37 [0.97,1.92]	0.73 [0.55,0.98]
Being head of household	1.30 [0.92, 1.85]	0.66 [0.39, 1.11]	1.23 [0.93,1.61]	0.97 [0.67,1.40]	0.59 [0.45,0.76]	1.24 [0.99,1.56]
Household size						
Living with 3–5 people	Ref	Ref	ref	ref	ref	Ref
Living alone or with 1–2 people	0.81 [0.66, 0.99]	1.18 [0.73, 1.92]	0.97 [0.76,1.23]	0.98 [0.78,1.23]	1.57 [1.25,1.97]	0.86 [0.68,1.09]
Living with 6 or more people	1.28 [1.01, 1.63]	0.66 [0.47, 0.93]	1.05 [0.84,1.31]	0.93 [0.79,1.09]	1.12 [0.93,1.36]	0.81 [0.68,0.95]
Religion						
Evangelical	Ref	Ref	ref	ref	ref	Ref
Catholic	1.36 [0.89, 2.09]	1.52 [1.00, 2.32]	0.89 [0.69,1.15]	0.93 [0.74,1.19]	1.15 [0.87,1.53]	1.09 [0.86,1.37]
Protestant	1.14 [0.83, 1.55]	0.98 [0.60, 1.62]	0.85 [0.64,1.14]	1.01 [0.81,1.27]	1.02 [0.76,1.36]	0.92 [0.68,1.23]
African Instituted Church /Other	0.91 [0.60, 1.39]	1.18 [0.69, 2.03]	1.04 [0.76,1.41]	0.87 [0.64,1.18]	1.09 [0.81,1.48]	0.78 [0.58,1.04]
Relative wealth						
Wealthier quintiles (vs Q1-Q2)	0.99 [0.77, 1.28]	0.45 [0.30, 0.67]	0.83 [0.66,1.05]	0.80 [0.65,0.99]	0.97 [0.75,1.26]	0.99 [0.82,1.20]

Finally, male participants were more likely to want to change their employment situation than female participants while older people were less likely to want this compared to younger people. There was no evidence of a statistically significant association with disability in the univariate, nor multivariable model (OR = 1.20 [0.90, 1.60]), similarly for the presence of functional difficulties (OR = 1.11 [0.80, 1.55]).

Male participants, older participants, those with salary, trade or another main source of income, and those from wealthier households were less likely to be in time-related underemployment than female participants, younger participants, those with farming as main source of income and those from poorer households. There was no evidence of significant association between disability and time-related underemployment (OR = 1.02 [0.78, 1.32]), results were similar for the presence of functional difficulties (OR = 0.86 [0.57,1.31]).

In terms of hours usually worked within a week, male participants, those with salary or trade as main source of income and those who were heads of household were less likely to work under 25 hours per week, compared to female participants, those with farming as main source of income and those who were not head of households. Older participants and those who lived in smaller households were more likely to work under 25 hours per week compared to younger participants and those who lived with 3–5 people. The association between time-related underemployment and disability was statistically non-significant (OR = 0.92 [0.70,1.21]). Results were similar for the association with functional difficulties (OR = 1.02 [0.68, 1.53]).

In terms of working days within a week, older people were more likely to work 6 or 7 days a week compared to younger people. Those with formal education and those with salary as main source of income were less likely to work 6–7 days a week compared to those with no formal education and those who reported farming as main source of income. There was no evidence of a statistically significant association between working 6–7 days a week and disability. Results remained similar when all other covariates were controlled for (OR = 0.95 [0.77, 1.18]); there was no evidence of an association with functional difficulties either (OR = 0.98 [0.72, 1.34]).

**Fig 1:**
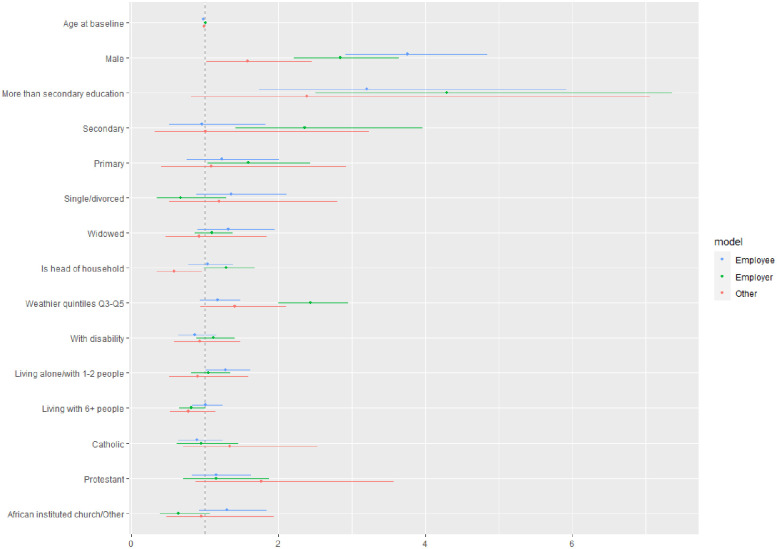
Associations with type of occupation—Results from univariate multinomial logistic regression models—Data are OR [95%CI].

The results of univariate multinomial regression models for associations between the covariates of interest and the type of engagement in the labour market are described in Fig 1. Being an independent worker without employees is used as the outcome of reference relative to which all the other types of employment are modelled.

Model results showed variations in type of occupation across age, sex, education levels, household size and relative wealth. For male participants, those with formal education and people from wealthier households, the relative odds of being employers versus independent workers were higher compared to, respectively, female participants, those with no formal education and those from poorer households. The relative odds of being employees versus independent workers were higher for younger people, those who attained more than secondary levels of education, who were from smaller households compared to, respectively, older people, those with no formal education and those living with 3 to 5 other people. Finally, the relative odds of being contributing family workers or dependent contractors versus independent workers without employees were higher for male participants compared to female participants, and lower for those who were heads of households compared to those who were not.

There was no evidence of significant association between disability status and type of occupation. This was also the case in multivariable models where all covariates were adjusted for (OR = 1.22 [0.95, 1.57], 0.86 [0.64, 1.15] and 0.98 [0.59, 1.62] for, respectively, employers, employees and other, versus independent worker). Results were similar when examining the association with functional difficulties (1.22 [0.89,1.67],0.68 [0.38,1.20],1.22 [0.59,2.53] for, respectively, employers, employees and other, versus independent worker).

## Discussion

In this large cohort of smallholder farmers in Western Kenya, almost 80% of the participants reported being engaged in the labour market and 17.2% reported having a disability. Having a disability was associated with a lower likelihood of labour market participation, regardless of age, sex, religion, education, marital status, or number of household members. Furthermore, when we looked at functional difficulties separately from anxiety and depression, the results were still similar. This finding is in line with our expectations and is broadly consistent with evidence from a limited number of previous studies from LMICs [42] which suggest that smallholder farmers with disabilities are disadvantaged in terms of labour market outcomes and should be targeted with programmatic initiatives to improve their participation and access to the labour market.

The prevalence of both disability and labour market outcomes varied considerably across sociodemographic groups. Similar to previous studies conducted in South Asia and sub-Saharan Africa [43], we found that women, older and widowed participants, those from poorer households and those who had never been to school were more likely to report having a disability. The relationship between gender, poverty and disability is well documented [44]. Although this study did not assess levels of assistive device use, evidence from similar settings suggests that the availability of good quality rehabilitation services for people with different types of impairments is low [45]. Improved availability of such services may help reduce self-reported functional difficulties and improve levels of participation. Unsurprisingly, having a functional difficulty was associated with depression and anxiety. The mechanisms that underlie the relationship between functional difficulties and depression/ anxiety are complex [46] and it would therefore be important for future analyses of data from this cohort to determine the impact of underlying depression on the functioning and well-being of farmers with different functional difficulties, and provide effective interventions. While we did not set out to examine a causal link in this cross-sectional analysis, farmers with disabilities in this rural African setting might benefit from interventions that integrate psychosocial support into economic empowerment initiatives.

The high proportion of participants in our study who reported being engaged in the labour market was consistent with another recent study from sub-Saharan Africa [47], in which, men were found to participate in the labour market to a significantly greater extent than women. Other studies of women’s participation in the labour market in sub-Saharan Africa, including studies in Kenya, show that women face greater challenges than men when it comes to accessing paid work [48–50], with resulting higher poverty rates [51, 52]. Women seeking to get into gainful employment often struggle to find a job not only in settings of high unemployment but also in areas where cultural norms and gender structures offer women less opportunities for participation in the labour market [53]. The difficulty can be compounded by having a disability [54] or loss of skill levels due to pregnancy-related absence from work [55]. In addition, the majority of employees in rural areas work in agriculture where participation often require land ownership but laws that accord women land rights are either absent or not sufficiently enforced across much of sub-Saharan Africa [56].

Our results are also consistent with those reported for a smaller cohort of 110 participants in a study conducted in northern Ghana, in which those who reported having higher levels of formal education and being from wealthier households were more likely to have a formal job [57]. This is in keeping with estimates from government reports which suggest that the bigger proportion of the labor force in developing countries is employed in informal jobs [58]. Some of the other previously documented determinants and dynamics of participation in the labour market were also seen in our cohort. In particular, we found that older age and living in a smaller household was strongly associated with working under 25-hours per week.

While our findings support previous research from other settings which has consistently found that functional difficulties and associated barriers to labour market participation are experienced differently by men and women [59], we did not find significant associations between having an informal job, the desire to change one’s employment situation, or time-related underemployment and disability. Our finding that women were more likely not to participate in the labour market than men is consistent with data from other studies in Kenya [60] and elsewhere on the African continent [47]. Longitudinal data that track smallholder farmers over time are needed to better understand the interplay of gender, disability and participation in the labour market in the longer-term.

The prevalence of disability in our sample of smallholder farmers was significantly higher than the overall prevalence of disability reported in the most recent Kenya population census (2.2%) [8]. While this finding may reflect our study setting in western Kenya, which has over the years been devastated by HIV and AIDS [61] and the high proportion of older, widowed women in our study sample, national censuses and community surveys in neighboring countries also report divergent disability prevalence estimates using comparable thresholds of the Washington Group Short Set of questions. For example, in Uganda the National Population and Housing Census of 2014 reported the overall prevalence of disability at only 12.4% [62]. However, data from a separate Demographic and Health Survey (DHS) conducted in 2016 found a much higher disability prevalence of 41% for adults aged 18 and over [63]. Another community survey based on the Washington Group Short Set (WGSS) of questions administered to a representative sample of Ugandans aged 50 and over who were either subsistence farmers or cattle keepers reported a disability prevalence of 21.7% [64]. In this large community survey, questionnaires were administered by trained health care workers, rather than enumerators of the type usually deployed during census data collection. This suggests that variations across census data and household surveys may in part be due to the form of questioning and the exposure and training of interviewers. The authors of the 2019 Kenya census report had also speculated that the low prevalence of disability could partly be because the interviewers did not administer the WGSS questions as trained [8].

Disability often goes largely unidentified (or undiagnosed) in resource-poor settings [65]. The relatively high prevalence of disability among farmers in this part of rural Kenya underscores the need to include screening and support for disability as a critically vital component of large-scale programmatic strategies to build sustainable livelihoods for people in resource-poor settings. Many people in rural Africa rely on subsistence agriculture to survive, and the International Labour Organization [37] has advocated a targeted focus on disability, gender, and labor rights for agricultural workers and a move away from the “one size fits all” approaches to inclusive development. Lessons need to be learned from the numerous and often small-scale economic empowerment programmes implemented in resource-poor settings by non-governmental organisations (NGOs) which have helped people with disabilities sustain or rebuild their livelihood assets and activities [66].

Our study should be considered within the context of four limitations. First, the measures assessed in our study are vulnerable to recall and social desirability bias. Data used for this study were self-reported by the smallholder farmers and it is known that self-reporting may lead to more socially desirable answers [67], which can result in an overestimation or under estimation of actual outcomes. For example, the highly subjective nature of the two questions used to assess depression and anxiety may have resulted in an underestimation due to social desirability bias [68]. In addition, and related to the above, disability in this setting is stigmatized [69], and farmers with some types of disabilities may not have wished to self-identify as persons with a disability, potentially leading to underreporting of disability. Second, our study includes only smallholder farmers aged 18 years and older, leaving adolescents who may be engaged in sorghum farming out of the sample. We used this criterion because 18 years is the legal age for adulthood in Kenya and except under specific circumstances and regulations set out in the relevant ILO Conventions, the employment of individuals aged younger than 18 years is not legal. Third, because this was a cross-sectional analysis, the findings cannot address the question of retention in employment, much less causal inferences about relationships between variables. Fourth, despite the large sample size and representativeness of the study population, our data were collected in one geographical region of the country and may be context-specific and thus cannot be directly generalizable to other regions with different contexts.

## Conclusions

These findings further our understanding of disability and participation in the labour market and can contribute to future guidelines for equitable participation in the labour market for people with disabilities. Our results highlight the continued need for research and policies to develop, evaluate, and implement labour market programmes in a manner that supports people with disabilities from diverse backgrounds. Future studies in other LMIC settings can better characterize how the different disability types are associated with labour force disadvantages and identify solutions that recognize the heterogeneity of smallholder farmers with disabilities, especially in resource-poor settings with high disability prevalence. There are no simple one-size-fits-all solutions to address the multifaceted labour force participation needs of smallholder farmers with disabilities, especially in settings of high unemployment. While study methodology limits our ability to generalize these findings to other populations, the implications of these data are important. Increased recognition and understanding of functional limitations among smallholder farmers is vital for the development of economic empowerment programmes aimed at increasing labour market participation. A greater understanding of these factors may potentially enhance the success of programmes directed toward people with disabilities and the labour market outcomes of vulnerable groups involved in these programmes.

## Supporting information

S1 File (DOCX)
