# Supplementary material for: Disability and labour market participation among smallholder farmers in Western Kenya
Source: PLoS One. 2024 Jul 5;19(7):e0306458. doi: 10.1371/journal.pone.0306458 (PMC11226002; doi:10.1371/journal.pone.0306458)
Supplement: S1 File — (DOCX) [file pone.0306458.s001.docx]

|  |  | Outcomes (ref: Independent worker without employees) | | | |
| --- | --- | --- | --- | --- | --- |
| Covariates |  | Employee | Employer | Other |  |
| Age at baseline |  | 0.98 [0.97, 0.99] | 1.01 [1.00, 1.01] | 0.99 [0.98, 1.01] |  |
| Sex | Male vs Female | 3.75 [2.91, 4.84] | 2.84 [2.21, 3.64] | 1.58 [1.02, 2.45] |  |
| Highest level of education (ref=Never went to school) | More than secondary | 3.20 [1.73, 5.92] | 4.29 [2.50, 7.36] | 2.39 [0.81, 7.06] |  |
|  | Secondary | 0.96 [0.51, 1.82] | 2.36 [1.41, 3.96] | 1.01 [0.32, 3.23] |  |
|  | Primary | 1.23 [0.75, 2.01] | 1.59 [1.04, 2.43] | 1.08 [0.40, 2.92] |  |
| Marital status (ref=Married/cohabiting) | Single/Divorced | 1.36 [0.88, 2.11] | 0.67 [0.35, 1.29] | 1.19 [0.51, 2.80] |  |
|  | Widowed | 1.32 [0.90, 1.95] | 1.09 [0.86, 1.38] | 0.92 [0.46, 1.84] |  |
| Head of household | Yes vs No | 1.04 [0.77, 1.39] | 1.29 [0.99, 1.68] | 0.58 [0.35, 0.96] |  |
| Relative wealth quintile | Wealthier quintiles Q3-Q5 vs Q1-Q2 | 1.17 [0.93, 1.48] | 2.43 [2.00, 2.95] | 1.40 [0.94, 2.10] |  |
| Disability status | With vs without disability | 0.86 [0.64, 1.15] | 1.11 [0.88, 1.40] | 0.93 [0.58, 1.48] |  |
| Household size (ref=living with 3-5 other people) | Living alone/ with 1-2 people | 1.28 [1.01, 1.62] | 1.05 [0.81, 1.35] | 0.90 [0.51, 1.59] |  |
|  | Living with 6+ people | 1.01 [0.82, 1.24] | 0.81 [0.65, 1.01] | 0.77 [0.52, 1.14] |  |
| Religion (ref=Evangelical) | Catholic | 0.89 [0.64, 1.24] | 0.95 [0.62, 1.45] | 1.34 [0.71, 2.53] |  |
|  | Protestant | 1.15 [0.82, 1.63] | 1.15 [0.71, 1.87] | 1.76 [0.87, 3.57] |  |
|  | African Instituted Church/Other | 1.30 [0.92, 1.84] | 0.64 [0.39, 1.06] | 0.95 [0.47, 1.94] |  |
